# Sodium portable tester for instantaneous natriuresis assessment: the SPOT-Na study

**DOI:** 10.1093/eschf/xvag166

**Published:** 2026-06-13

**Authors:** Thomas Goria, Anaïs Curtiaud, Sabrina Garnier Kepka, Jean-Jacques Von Hunolstein, Hamid Merdji, François Sauer, Julien Demiselle, Antonin Trimaille, Nans Florens

**Affiliations:** Nephrology, Dialysis and Transplantation Department, Strasbourg University Hospitals, 67091 Strasbourg, France; Service de Médecine Intensive-Réanimation, Fédération Hospitalo-Universitaire TARGET, Hôpitaux Universitaires de Strasbourg, Strasbourg, France; INSERM UMR 1260, Regenerative Nanomedicine, FMTS, Strasbourg, France; Emergency Department, Strasbourg University Hospitals, Strasbourg, France; Department of Cardiovascular Medicine, Nouvel Hôpital Civil, Strasbourg University Hospital, Strasbourg, France; Service de Médecine Intensive-Réanimation, Fédération Hospitalo-Universitaire TARGET, Hôpitaux Universitaires de Strasbourg, Strasbourg, France; INSERM UMR 1260, Regenerative Nanomedicine, FMTS, Strasbourg, France; Department of Cardiovascular Medicine, Nouvel Hôpital Civil, Strasbourg University Hospital, Strasbourg, France; Service de Médecine Intensive-Réanimation, Fédération Hospitalo-Universitaire TARGET, Hôpitaux Universitaires de Strasbourg, Strasbourg, France; INSERM UMR 1260, Regenerative Nanomedicine, FMTS, Strasbourg, France; Department of Cardiovascular Medicine, Nouvel Hôpital Civil, Strasbourg University Hospital, Strasbourg, France; Research Unit UR3074 – Translational Cardiovascular Medicine, University of Strasbourg, Strasbourg, France; Nephrology, Dialysis and Transplantation Department, Strasbourg University Hospitals, 67091 Strasbourg, France; Laboratoire d'Immuno Rhumatologie Moléculaire, INSERM UMR_S1109, FHU TARGET, FMTS, Université de Strasbourg, 67000 Strasbourg, France; INI-CRCT Network, F-CRIN, 54500 Vandoeuvre-lès-Nancy, France

**Keywords:** Urinary sodium, Point-of-care testing, Natriuresis, Diuretic resistance, Acute heart failure, Decongestion

Effective decongestion is a cornerstone of therapy in volume overload states, including acute heart failure (AHF). Inadequate natriuresis despite loop diuretics is associated with persistent congestion and worse outcomes. The Heart Failure Association of the European Society of Cardiology recommends early urinary sodium measurement within 1–2 h of diuretic administration, with prompt therapy intensification when levels remain low.^1,2^ The PUSH-AHF trial subsequently validated a 70 mmol/L threshold for triggering diuretic escalation and further underscored the potential of natriuresis-guided therapy.^3^ Yet widespread adoption of this strategy remains limited by delays inherent to laboratory-based urinary sodium measurements and the absence of convenient bedside tools.

The SPOT-Na study evaluated a portable point-of-care sodium metre (LAQUAtwin Na^+^, Horiba, Japan) for instantaneous urinary sodium analysis. We conducted a prospective, single-centre study enrolling adults requiring urinary sodium measurement as part of routine clinical care across cardiology, nephrology, emergency, and intensive care units. The study was approved by the institutional ethics committee (reference R25-002), and informed consent was obtained from all participants. Urinary sodium was measured simultaneously by the hospital laboratory (potentiometry using an ion-selective electrode on the Cobas® pro ISE analytical unit, Roche Diagnostics GmbH, Mannheim, Germany) and by the handheld LAQUAtwin Na^+^ device at the bedside. Primary endpoints were agreement between methods (Pearson correlation, Bland–Altman bias, and limits of agreement) and difference in time-to-result.

A total of 157 patients were analysed (mean age 64 years, 58% men) from cardiology (*n* = 48), nephrology (*n* = 54), emergency (*n* = 18), and intensive care (*n* = 37) units, with frequent heart failure (46%) and acute kidney injury (29%). The point-of-care metre required only .3 mL of urine and yielded a result in under 10 s, whereas laboratory processing required a median of 104 min (interquartile range 77–139) (*P* < .0001).

Pearson correlation between methods was *r* = .92 (*R*^2^ = .85) (*Figure 1A*). The mean bias was −10 mmol/L, with the LAQUAtwin device reading systematically lower than the laboratory. Bland–Altman analysis confirmed this modest underestimation with 95% limits of agreement of −60 to +34 mmol/L. Performance was acceptable across the mid-range of 50–100 mmol/L but diverged at extremes. The device tended to underestimate at very high concentrations (>100–150 mmol/L), while comparison below 20 mmol/L was constrained by the laboratory’s reporting floor; all samples below this floor were correctly flagged by the LAQUAtwin.

**Figure 1:**
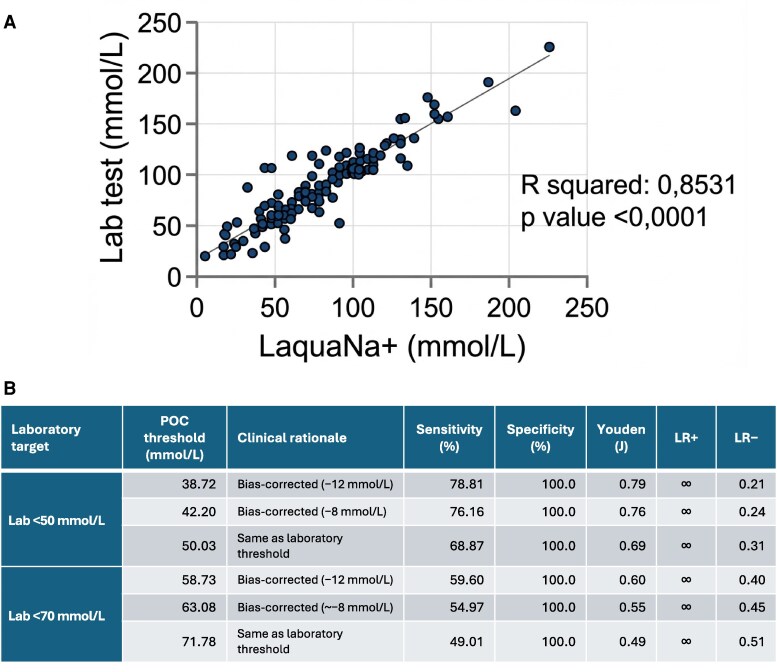
Diagnostic performance of the LAQUAtwin Na^+^ point-of-care device for urinary sodium measurement. (A) Scatter plot showing the Pearson correlation between point-of-care (LAQUAtwin Na^+^, *x*-axis) and laboratory urinary sodium measurements (*y*-axis), each point representing a paired sample (*n* = 157). Values below 20 mmol/L were censored because the laboratory does not report exact measurements below this threshold (*n* = 21 samples affected). *R*^2^ = .85, *P* < .0001. (B) Diagnostic performance (sensitivity, specificity, Youden index J, and likelihood ratios LR+ and LR−) of clinically relevant point-of-care sodium thresholds for detecting laboratory-defined insufficient natriuresis (<50 mmol/L or <70 mmol/L). Thresholds are presented either matching the laboratory cut-off directly or corrected for the known systematic negative bias of the device (8–12 mmol/L lower than laboratory values)

Because the LAQUAtwin systematically underestimates urinary sodium by 8–12 mmol/L, diagnostic performance was assessed against both unadjusted laboratory thresholds (50 and 70 mmol/L) and bias-corrected thresholds (*Figure 1B*). Without correction, sensitivity at the 50 and 70 mmol/L thresholds were 68.9% and 49.0%, respectively, with specificity of 100% in both cases. After bias correction, applying LAQUAtwin thresholds of 38–42 mmol/L for a laboratory target of 50 mmol/L, or 58–63 mmol/L for 70 mmol/L, sensitivity improved markedly to 76.2%–78.8% and 55.0%–59.6%, respectively, while 100% specificity was preserved throughout.

These findings carry direct clinical implications. The ability to obtain urinary sodium results in seconds at the bedside may transform natriuresis-guided diuretic titration in AHF. Clinicians can rapidly identify diuretic non-responders (for instance, a spot urine sodium below 50 mmol/L within 2 h of loop diuretic administration) and escalate therapy during the same clinical encounter rather than awaiting laboratory results for nearly 2 h. This real-time feedback addresses a key operational shortcoming identified in the PUSH-AHF trial. The ease-of-use design also suggests integration into nursing-led natriuresis monitoring protocols, consistent with findings from the EASY-HF and ENACT-HF studies.^4,5^

Several limitations merit acknowledgement. Individual variability was substantial (limits of agreement ±30–50 mmol/L in some pairs), underscoring that the device functions best as a rapid screening tool for identifying low- or high-natriuresis states rather than as a substitute for precise laboratory quantification in threshold-sensitive decisions. The monocentric design and heterogeneous population limit generalizability. Importantly, whether point-of-care natriuresis monitoring translates into improved clinical outcomes (including length of stay or survival) remains to be established in a randomized controlled trial.

In conclusion, the SPOT-Na study demonstrates that handheld urinary sodium testing provides rapid, clinically acceptable natriuresis assessment with strong correlation to laboratory reference values and a reduction in time-to-result of over 2 h. Accounting for the device’s predictable negative bias through adjusted decision thresholds preserves excellent specificity while meaningfully improving sensitivity. This point-of-care approach may help translate natriuresis-guided therapy into routine AHF practice, particularly in settings where rapid urinary sodium measurement is not currently feasible.

## Declarations

### Data Availability

The data underlying this article will be shared on reasonable request to the corresponding author.
